# P-856. Beta Lactams as Oral Step Down Therapy in the Treatment of Uncomplicated Gram Negative Bacteremia: A Systematic Review and Meta Analysis

**DOI:** 10.1093/ofid/ofae631.1048

**Published:** 2025-01-29

**Authors:** Michael Dore, Ryan Duffy, Lily Huang, Laura Caputo, Sarah Cantrell, Blair Glasgo

**Affiliations:** Department of Veterans Affairs, Durham, North Carolina; Department of Veterans Affairs, Durham, North Carolina; Department of Veterans Affairs, Durham, North Carolina; Department of Veterans Affairs, Durham, North Carolina; Duke University, Durham, North Carolina; Department of Veterans Affairs, Durham, North Carolina

## Abstract

**Background:**

Gram-negative bacteremia (GNB) treatment is initially intravenous but is often transitioned to oral antibiotics once stable. This decision is based on culture sensitivity, antibiotic bioavailability, and antibiotic side effect profile. Historically, fluoroquinolones (FQ) or trimethoprim-sulfamethoxazole (TMP-SMX) are selected over beta-lactams (BL) due to concerns about pharmacokinetics and bioavailability. In 2019, a systematic review and meta-analysis compared the use of BL for step-down therapy vs FQ and TMP-SMX was inconclusive, and the implementation of BL has not been widely adopted. Since then, several moderate-sized studies have evaluated this question but are limited by sample size. We performed a systematic review with meta-analysis evaluating BL in the treatment of uncomplicated GNB.

**Figure 1:**
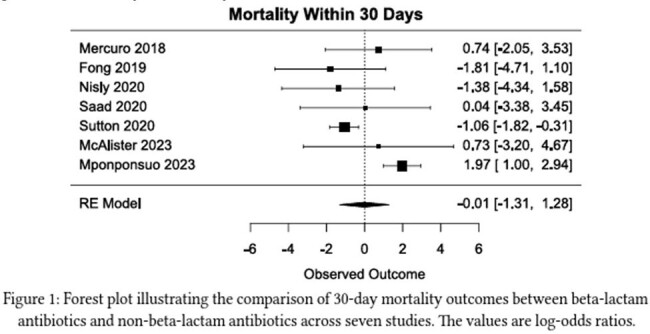
30 Day Mortality

Forest plot illustrating the 30 day mortality comparison between beta lactams and FQ/TMP-SMX

**Methods:**

The MEDLINE, Embase, and Web of Science databases were searched from inception to August 2023 for published articles related to bacteremia, BL antibiotics, and FQ or TMP-SMX antibiotics. Inclusion criteria included adults aged 18 years or older with uncomplicated GNB treated with IV antibiotics initially and transitioned to appropriate oral antibiotics based on sensitivity testing. Uncomplicated infections were defined as having definitive source control within 48 hours.

**Figure 2:**
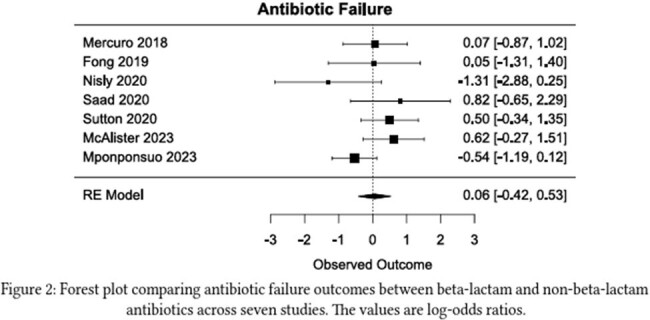
Antibiotic Failure Rate

Forest plot illustrating the antibiotic failure rate between beta lactams and FQ/TMP-SMX

**Results:**

The search yielded a total of 5029 citations. 1598 were removed as duplicates. 3431 titles and abstracts were screened with 3411 excluded. 20 full texts were retrieved and evaluated for eligibility; 13 were excluded and 7 were reviewed. A total of 7306 patients were included in the study with twice as many as the FQ/TMP-SMX vs BL group (4879 vs 2407). The average age (69 vs 71), percent male (51 vs 52%), percent urinary source (75 vs 77%), duration of IV antibiotics (4.2 vs 4.6), Pitt bacteremia score (1.1 vs 1.4) and Charleston comorbid index (2 v 2.2) were similar in each group. There was no statistical difference between the 30-day mortality -0.01 (95% CI -1.31 to 1.28) or antibiotic failure: 0.06 (95% CI -0.42 to 0.53)

**Conclusion:**

BL are non-inferior to FQ or TMP-SMX as oral step-down therapy in the treatment of uncomplicated GNB . The individual decision to use a FQ, TMP-SMX or BL should be based on the patients' comorbid conditions, potential drug-drug interactions, cost, and potential for adverse events.

**Disclosures:**

**All Authors**: No reported disclosures

